# Nordihydroguaiaretic Acid from Creosote Bush (*Larrea tridentata*) Mitigates 12-*O*-Tetradecanoylphorbol-13-Acetate-Induced Inflammatory and Oxidative Stress Responses of Tumor Promotion Cascade in Mouse Skin

**DOI:** 10.1093/ecam/nep076

**Published:** 2011-06-05

**Authors:** Shakilur Rahman, Rizwan Ahmed Ansari, Hasibur Rehman, Suhel Parvez, Sheikh Raisuddin

**Affiliations:** ^1^Department of Medical Elementology and Toxicology, Hamdard University, New Delhi 110062, India; ^2^Department of Pharmaceutical Sciences, Medical University of South Carolina, Charleston, SC 29425, USA; ^3^Department of Neurophysiology, Leibniz Institute for Neurobiology, Brenneckestrasse 6, Magdeburg D-39118, Germany

## Abstract

Nordihydroguaiaretic acid (NDGA) is a phenolic antioxidant found in the leaves and twigs of the evergreen desert shrub, *Larrea tridentata* (Sesse and Moc. ex DC) Coville (creosote bush). It has a long history of traditional medicinal use by the Native Americans and Mexicans. The modulatory effects of topically applied NDGA was studied on acute inflammatory and oxidative stress responses in mouse skin induced by stage I tumor promoting agent, 12-*O*-tetradecanoylphorbol-13-acetate (TPA). Double TPA treatment adversely altered many of the marker responses of stage I skin tumor promotion cascade. Pretreatment of NDGA in TPA-treated mice mitigated cutaneous lipid peroxidation and inhibited production of hydrogen peroxide. NDGA treatment also restored reduced glutathione level and activities of antioxidant enzymes. Elevated activities of myeloperoxidase, xanthine oxidase and skin edema formation in TPA-treated mice were also lowered by NDGA indicating a restrained inflammatory response. Furthermore, results of histological study demonstrated inhibitory effect of NDGA on cellular inflammatory responses. This study provides a direct evidence of antioxidative and anti-inflammatory properties of NDGA against TPA-induced cutaneous inflammation and oxidative stress corroborating its chemopreventive potential against skin cancer.

## 1. Introduction

**Figure 1:**
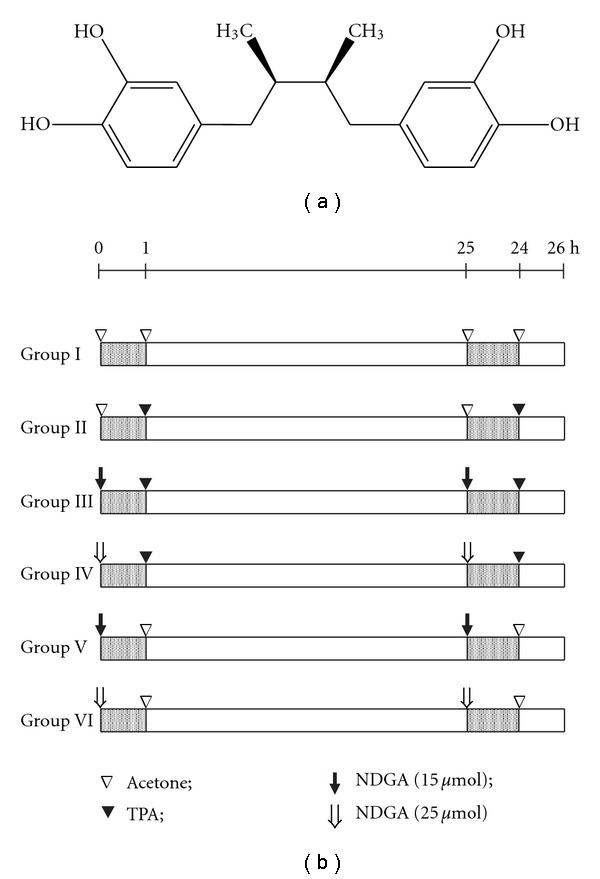
(a) Chemical structure of NDGA and (b) schematic representation of dosing schedule in different groups of animals. Animals were divided into six groups I–VI (*n* = 6). Control animals (Group I) were treated with topical application of acetone (Ac, 100 *μ*l). Group II animals were treated with 10 nmol of TPA/100 *μ*l of acetone. NDGA (15 *μ*mol/100 *μ*l of acetone) 1 h before TPA (10 nmol/100 *μ*l of acetone) treatment was applied to Group III animals. Group IV animals were treated with topical application of NDGA (25 *μ*mol/100 *μ*l of acetone) 1 h before TPA (10 nmol/100 *μ*l of acetone) treatment. Groups V and VI animals received topical application of 15 and 25 *μ*mol NDGA in 100 *μ*l acetone, respectively. After 24 h the same doses of NDGA or acetone were applied 1 h prior to the second TPA application (10 nmol/100 *μ*l acetone). Animals were sacrificed by cervical dislocation after 1 h of second TPA application. The skin application area was ~6 cm^2^.

Nordihydroguaiaretic acid (NDGA), 2,3-dimethyl-l,4-*bis *(3,4-dihydroxyphenyl) butane (Figure 1(a)) is a plant lignan derived from the leaves and twigs of shrub creosote bush, *Larrea tridentata* (Sesse and Moc. ex DC) Coville; family Zygophyllaceae. Creosote bush dominates areas of the desert southwest in the USA and Northern Mexico, as well as some areas of Argentina [1]. It is also known as chaparral and greasewood in the USA and gobernadora (governess) and hediondilla (little smelly one) in Mexico. This shrub has a long history of traditional medicinal use for a variety of health problems by the Native Americans and Mexicans [1]. Chaparral tea has been used in the folk medicine for the treatment of more than 50 ailments including infertility, tuberculosis, arthritis, diabetes, kidney and gallbladder stones, pain and inflammation [1]. The concentration of NDGA in *L. tridentata* leaves is in the range of 5%–10% of dry weight [1]. NDGA is a recognized inhibitor of lipoxygenase (LOX) and has antioxidant and free radical scavenging properties [2, 3]. Protective effect of NDGA has been observed against hepatic and renal toxicities induced by ferric-nitrilotriacetate (Fe-NTA) [4]. It also showed protective effect against ozone-induced tyrosine nitration in lungs [2], potassium dichromate-induced oxidative stress and nephrotoxicity [3] and streptozotocin-induced diabetic nephropathy [5]. Besides its antioxidant activity, NDGA has several other properties, which are of potential use for humans such as it acts as an enzyme inhibitor [6], antimicrobial agent [7, 8], potential vaso- and brancho-dilating agent [9] and antimutagenic agent [10].

In contrast with the beneficial effects described above for NDGA, adverse effects for chaparral containing products have also been observed when ingested in high doses [1, 11]. Therefore, NDGA was removed from the generally regarded as safe (GRAS) list of the Food and Drug Administration (FDA) in 1968 following several reports of nephrotoxicity and hepatotoxicity in humans [12, 13].

NDGA has shown chemopreventive activity in various models of carcinogenesis [4, 14]. It restores expression of silenced E-cadherin gene in human breast cancer cell lines and in xenografts [15] and reduces the lung adenoma multiplicity in urethane-treated mice [14]. NDGA suppresses benzoyl peroxide (BPO) and 12-*O*-tetradecanoylphorbol-13-acetate (TPA)-induced ornithine decarboxylase (ODC) activity and tumor promotion in murine skin [16–18]. Since metabolic products of LOX pathway play an important role in tumor promotion, anti-LOX activity of NDGA has been identified as one of the possible mechanisms of its chemopreventive effect [17].

Skin cancer is a major health problem associated with mortality and morbidity. Chemoprevention is one of the many favorite current approaches applied to ameliorate the occurrence of skin cancer [19]. A number of chemopreventive agents have been tested for their efficacy in mitigating the responses of promotion stage of skin cancer development, as this stage is observed to be susceptible to reversibility [20]. TPA is the most widely used tumor-promoting agent in experimental skin carcinogenesis studies. TPA treatment results in oxidative stress and a decrease in skin antioxidants [21]. Recent studies have suggested that the double TPA application model is appropriate to predict the inhibitory potential of a test compound against tumor promotion in skin [22, 23].

Certain natural products, fruits, vegetables, common beverages and several herbs are rich sources of pharmacologically active chemicals with diversified activities [22–25]. Due to low toxicity and relative safety natural compounds have promising chemopreventive potential with capacity to intervene more than one critical pathway [26, 27]. Therefore, in the present study we tested the effect of NDGA against inflammatory and oxidative stress responses induced by double TPA application in mouse skin. These inflammatory and oxidative stress responses empirically represent typical stage I tumor promotion events.

## 2. Methods

### 2.1. Animals

Swiss albino female mice (25 ± 2 g) were provided by the Central Animal House Facility of the University and maintained under standard laboratory conditions (temperature 25 ± 2°C; photoperiod of 12 h) on a commercial pellet diet and water *ad libitum*. The study protocols were approved by the Institutional Animals Ethics Committee (project #266).

### 2.2. TPA Application Protocol and Skin Tissue Processing

Dorsal skin of animals was shaved using surgical clippers (Oster Professional Products, McMinnville, TN, USA) two days before commencement of treatment. NDGA (Sigma-Aldrich Co., St Loius, MO, USA; 15 and 25 *μ*mol in 100 *μ*l of acetone, BDH, Mumbai, India) was topically applied onto the shaved area of dorsal skin 1 h before application of TPA (Sigma; 10 nmol in 100 *μ*l of acetone). The same doses of NDGA and TPA or acetone were applied at an interval of 24 h. The timing (24 h apart) of double TPA application produced the same level of oxidative stress as produced in twice-weekly application protocol (24 h–72 h) used in skin tumor promotion studies [23]. A schematic representation of dosing schedule is given in Figure 1(b). Animals were sacrificed by cervical dislocation under mild anesthesia 1 h after the second TPA application and tissue from the dorsal area was surgically removed. A piece of skin was preserved in 10% neutral buffer formalin for histological investigation. Homogenates were prepared in chilled phosphate buffer (0.1 M, pH 7.4) using Polytron homogenizer (Kinematica, Inc., Bohemia, NY, USA). The homogenized tissue was centrifuged at 10 500 g for 30 min at 4°C to obtain the post-mitochondrial supernatant (PMS). 

### 2.3. Myeloperoxidase Activity and Edema Formation

The Myeloperoxidase (MPO) activity was determined by the method of Bradley et al. [28] and expressed as units/min/mg protein. One unit of MPO activity was defined as that degrades 1 *μ*mol of peroxide per min. The edema formation in skin of various groups of animals was measured using the skin punch method of Huang et al. [29] and result is expressed as mg/punch.

### 2.4. Lipid Peroxidation, Hydrogen Peroxide Production and Xanthine Oxidase Activity

Lipid Peroxidation (LPO) was measured using the procedure of Uchiyama and Mihara [30] and the rate of LPO was determined as nmol of thiobarbituric acid reactive substances (TBARS) formed/h/g of tissue. Hydrogen peroxide (H_2_O_2_) production was measured by the method of Pick and Keisari [31] and calculated as nmol H_2_O_2_/h/g tissue. The xanthine oxidase (XO) activity was measured by the method of Stripe and Della-Corte [32] and results are expressed as *μ*g uric acid formed/mg protein.

### 2.5. Skin Antioxidants

Glutathione (GSH) was measured by the method of Jollow et al. [33] and result is expressed as nmol GSH/g tissue. Activities of glutathione peroxidase (GPx) were measured by Lawrence and Burk [34] and glutathione reductase (GR) was measured according to the method of Carlberg and Mannervik [35] and calculated as nmol nicotinamide adenine dinucleotide phosphate (NADPH, Sigma) oxidized/min/mg protein. Glutathione *S*-transferase (GST) activity was measured by the method of Habig et al. [36] and expressed as nmol 1-chloro-2,4-dinitrobenzene (CDNB, Sigma) conjugates formed/min/mg protein. Catalase (CAT) activity was assayed by the method of Claiborne [37] and expressed as nmol H_2_O_2_ consumed/min/mg protein. Superoxide dismutase (SOD) activity was measured according to the method of Marklund and Marklund [38] and expressed as units/mg protein. One unit of SOD was defined as the enzyme activity that inhibited autoxidation of pyrogallol (Sigma) by 50%.

### 2.6. Histology

The formalin fixed skin samples were dehydrated with graded ethanol (BDH), and embedded in paraffin (Hi-Media Labs, Mumbai, India) after rinsing with tap water. The samples were cut by microtome at 5 *μ*m and mounted on glass slides. The sections were dewaxed using xylene (BDH) solution (mixture of isomers) and graded ethanol, and stained with hematoxylin and eosin (Hi-Media Labs). Inflammatory cell (peripheral mononuclear cells) infiltration and intercellular edema (accumulation of fluid between the epidermal cells) were scored as slight (+), moderate (++) or severe (+++), while (−) indicates absence of a significant change. The number of nucleated cell layers in the epidermis was determined by counting the average numbers at five randomly selected locations per slide.

### 2.7. Statistical Analysis

One-way analysis of variance (ANOVA) was applied to determine significant differences in results of various groups. *P*-values < .05 were considered significant. Subsequently, Tukey's *t*-test was applied for analyzing the significance between different treatment groups. The values are expressed as means ± SE.

## 3. Results

### 3.1. Inflammatory Responses

**Figure 2:**
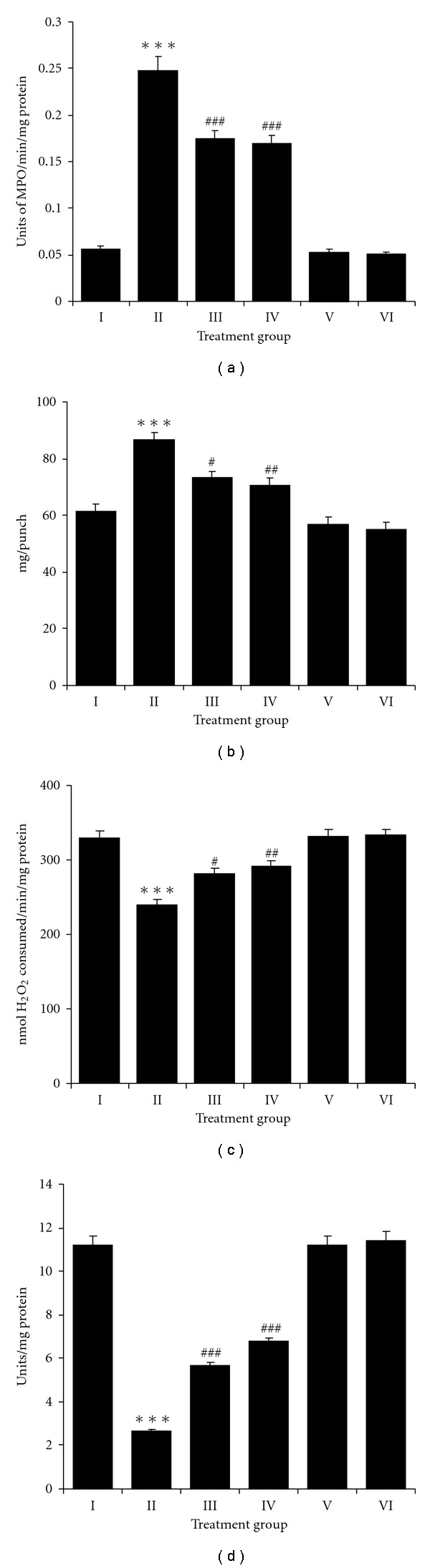
Effect of NDGA on TPA induced (a) MPO activity, (b) edema formation, (c) CAT and (d) SOD activity in mouse skin from different treatment groups. Values are expressed as means ± SE (*n* = 6) of units of MPO/min/mg protein, cutaneous edema as mg/punch, catalase activity as nmol H_2_O_2_ consumed/min/mg protein and SOD activity as units/mg protein. Significant differences are indicated by ***P < .001 when compared with control animals (Group I) and ^#^ *P* < .05, ^##^ *P* < .01 and ^###^ *P* < .001 when compared with TPA-treated animals (Group II).

Double TPA application at a dose of 10 nmol each with a 24 h interval caused significant increase in the activity of MPO (*P* < .001) in TPA treated group when compared with control group (Figure 2(a)). Animals with NDGA treatment showed significant reduction (*P* < .001) in MPO activity when compared to TPA-treated group. NDGA alone at either 15 or 25 *μ*mol caused no significant changes in the above parameters compared to controls. Double application of TPA 24 h apart caused significant edema formation when compared with Group I animals (*P* < .001) (Figure 2(b)). Animals of Groups III and IV which were treated with 15, 25 *μ*mol of NDGA, respectively, showed a significantly reduced (*P* < .05 and .01) edema response when compared with TPA-treated animals (Group II).

### 3.2. LPO, H_2_O_2_ Production and XO Activity

**Figure 3:**
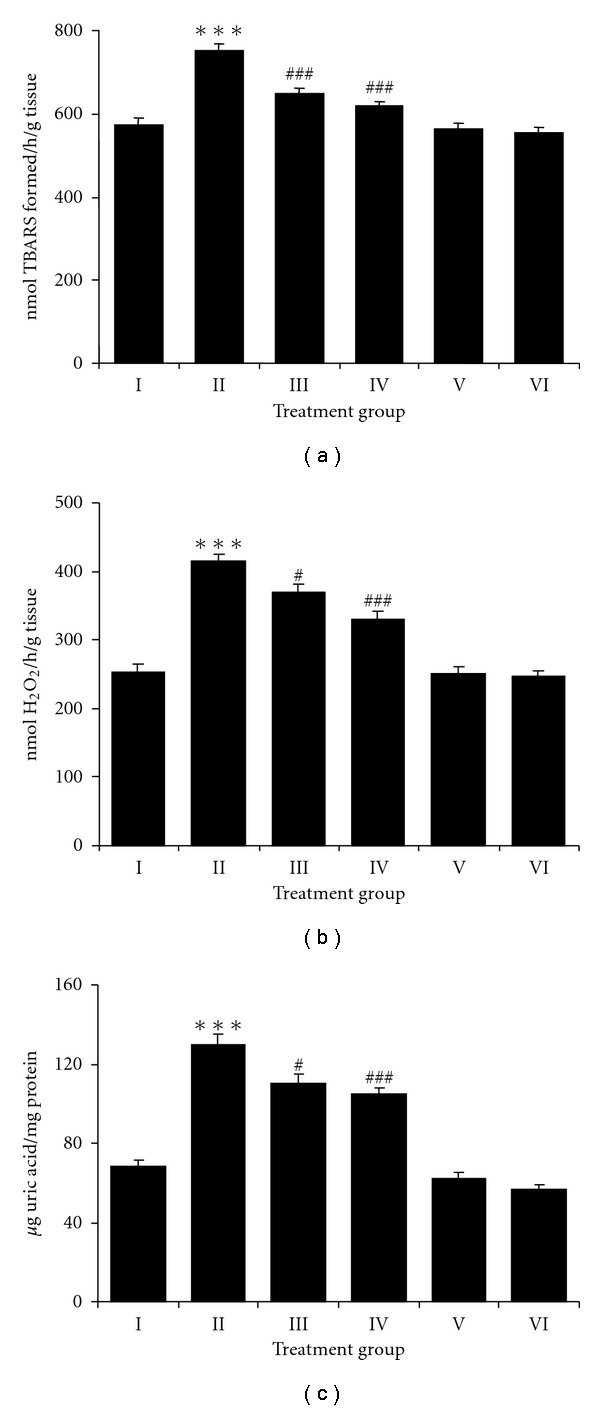
Effect of NDGA on TPA-induced (a) lipid peroxidation, (b) H_2_O_2_ production and (c) xanthine oxidase activity in skin of mice from different treatment groups. Values are expressed as means ± SE (*n* = 6) of nmol TBARS formed/h/g tissue, nmol H_2_O_2_/h/g tissue and *μ*g uric acid/mg protein, respectively. Significant differences are indicated by ***P < .001 when compared with control animals (Group I) and ^#^ *P* < .05 and ^###^ *P* < .001 and when compared with TPA-treated animals (Group II).

TPA treated animals showed a significant increase (*P* < .001) in the levels of LPO (Figure 3(a)), H_2_O_2_ production (Figure 3(b)) and XOD activity (Figure 3(c)) in skin when compared with control group. Groups III and IV (NDGA treatment) animals showed a significant decrease in all these parameters (*P* < .05– .001). NDGA alone at both the doses (15 or 25 *μ*mol) caused no significant changes in any of the above parameters compared to controls.

### 3.3. Skin Antioxidants

**Figure 4:**
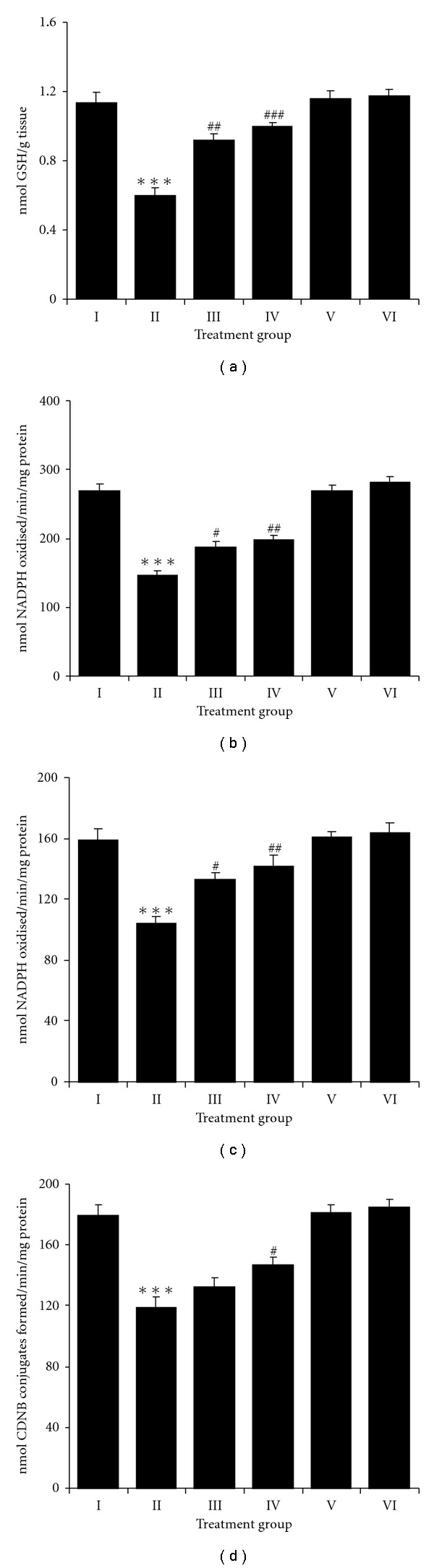
Effect of NDGA on TPA induced (a) GSH level, (b) GPx, (c) GR and (d) GST activity in mouse skin from different treatment groups. Values are expressed as means ± SE (*n* = 6) of GSH/g tissue, GPx and GR activities as nmol NADPH oxidized/min/mg protein, GST activity as nmol CDNB conjugates formed/min/mg protein. Significant differences are indicated by ***P < .001 when compared with control animals (Group I) and ^#^ *P* < .05, ^##^ *P* < .01 and ^###^ *P* < .001 and when compared with TPA-treated animals (Group II).

The double application of TPA caused a significant decrease in the activities of all the glutathione metabolizing enzymes such as GPx (*P* < .001), GR (*P* < .001), GST (*P* < .001) compared to control animals (Figures 4(b)–4(d)). Application of NDGA (15 *μ*mol) in TPA-treated animals showed a significant increase in the activities of GPx (*P* < .05) and GR (*P* < .05). However, no significant increase in the activity of GST is observed. Similarly, higher dose of NDGA (25 *μ*mol) showed significant increase in the activities of GPx (*P* < .01), GR (*P* < .01) along with a significant increase in GST (*P* < .05) activity (Figures 4(b)–4(d)). GSH data also showed a significant decrease (*P* < .001) in TPA-treated group. NDGA along with TPA at both low and high doses caused a significant increase in the activities of GSH (*P* < .01 and *P* < .001) (Figure 4(a)), Concomitant to decrease in glutathione cycle enzymes a significant decrease in the activities of other antioxidant enzymes such as CAT (*P* < .001) and SOD (*P* < .001) was also observed in the TPA-treated animals (Group II) compared to control animals (Figures 2(c) and 2(d)). Animals with NDGA treatment (15 and 25 *μ*mol) showed a significant increase in the activities of SOD and CAT when compared to TPA-treated animals. Changes in the above parameters were not significant when NDGA applied alone at either 15 or 25 *μ*mol as compared to controls.

### 3.4. Histological Findings

**Figure 5:**
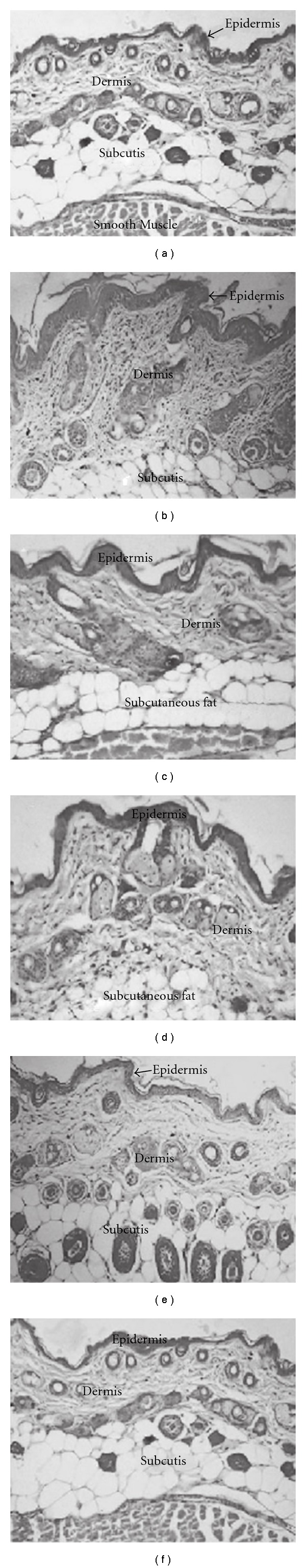
Effect of NDGA on TPA induced morphological changes as observed by H & E, ×100. Representative pictures are shown. (a) Photomicrograph of skin section from acetone treated control animal showing normal skin structure having well defined epidermal and dermal layers. Epidermis is just 2-3 cells thick. Superficial dermis does not show any edema or PMN infiltration; (b) double dose of TPA treatment showing skin with irregular epidermal thickening, severe dermal edema and infiltration of PMNs in upper dermis; (c) mice treated with NDGA (15 *μ*mol) before topical application of TPA showing moderate epidermal thickening moderate dermal edema and scattered dermal infiltrates; (d) section of skin treated with higher dose of NDGA (25 *μ*mol) before TPA application showing mild epidermal thickening, occasional infiltration of leukocytes and mild edema; and (e) and (f) sections of skin from mice treated with NDGA 15 and 25 *μ*mol, respectively, followed by acetone application showing normal skin structure. Detail of histological observations is also given in Table 1.

Double application of TPA caused marked histological alteration such as increase in epidermal cell layer, large number of infiltrating polymorphonuclear leukocytes (PMNs) and intercellular edema in skin showing inflammatory responses in the tissue (Figures 5(b) Table 1). Control animals showed a normal tissue structure (Figure 5(a)). However, Groups III and IV animals, which were treated with NDGA (15 or 25 *μ*mol) before application of TPA showed mitigation of the above inflammatory effects (Figures 5(c) and 5(d); Table 1). Animals of Groups V and VI, which were treated with NDGA (15 and 25 *μ*mol, resp.) showed normal skin structure with no remarkable histological changes over the control skin (Figures 5(e) and 5(f)).

## 4. Discussion

TPA is a typical tumor-promoting agent used extensively in experimental carcinogenesis studies. Topical application of TPA induces biochemical alterations and cellular and histological changes leading to skin tumor promotion [21]. All these alterations in skin are defined as possible markers of tumor promotion and therefore are the targets of chemopreventive agents [21, 22]. Tumor promotion is also accompanied by inflammation and oxidative stress. It is likely that a compound with strong anti-inflammatory and/or antioxidant activities may be a good candidate for intercepting the promotional events in tumorigenesis [39]. In this study, we observed that NDGA was capable of modulating many of the TPA-induced inflammatory and oxidative stress responses.

Although a single TPA application is sufficient to induce marker responses of tumor promotion events, the double application of TPA has more remarkable effect as it induces two distinguishable biochemical events, namely, “priming” and “activation” [23, 40]. The first event is characterized by recruitment of inflammatory cells such as polymorphonuclear leukocytes by chemotactic factors to inflammatory regions and the second event involves activation of neutrophils or other oxidant-producing cells including keratinocytes. The final outcome of both the processes is a massive inflammatory response, which overwhelms the endogenous anti-inflammatory and antioxidant milieu of the cells. Thus, it is more appropriate to test the efficaciousness of a target chemopreventive agent in double TPA application protocol. Results of the present study indicate that double application of TPA causes an increase in MPO activity and skin edema formation, which are hallmarks of inflammatory response. Increase in the activity of MPO may be due to large number of infiltrating PMNs in TPA-treated group. Pretreatment of NDGA inhibited these inflammatory biomarkers. Accumulation of inflammatory cells (e.g., PMNs) as a result of TPA application is responsible for ROS generation, which plays an important role in skin tumor promotion [20].

It is generally accepted that TPA acts as a strong promoting agent through oxygen-mediated mechanism and that oxygen radicals are the critical components of the tumor promotion process [41]. Double TPA application caused increased H_2_O_2_ production, XO activity and LPO whereas pretreatment with NDGA markedly inhibited these effects. Murakami et al. [42] suggested that decreased levels of H_2_O_2_ might be attributable to the inhibition of O_2_^•−^ as a function of SOD or by a nonenzymatic mechanism.

TPA application caused increase in XO activity, which may be due to TPA-dependent induction of xanthine dehydrogenase (XD) synthesis and conversion of XD to XO [41]. XO activity is also correlated with the degree of hyperplasia [43]. We also observed a close correlation between increase in XO activity and number of epidermal cell layers in histological analysis. NDGA treatment effectively inhibited XO activity and showed marked improvement in the histology of treated skin. Dhawan et al. [44] suggested that LPO might be one of the factors contributing to tumor formation. During oxidative stress, MDA and/or other aldehydes are formed which react with amino acids and DNA and introduce cross linkages between proteins and nucleic acids resulting in alteration in replication and transcription [44]. LPO is also typically associated with tumor promotion stage. The protective effect of NDGA against TPA-induced LPO reported here along with other modulatory effects on inflammatory and oxidative stress responses supports chemopreventive potential of NDGA.

Antioxidant enzymes such as CAT, SOD and GPx counteract and regulate overall ROS level to maintain physiological homeostasis [41]. Application of NDGA at both doses ameliorated TPA-mediated oxidative stress including restoration of these enzymes. Level of GSH was also restored suggesting a multiple protective effect for NDGA. GSH is involved in maintaining intracellular redox status by regulating and controlling oxidative stress [45]. GSH directly scavenges ROS and its loss is associated with an augmented pro-inflammatory response [46].

NDGA treatment before TPA application caused improvement in skin histology such as changes in degree of PMN infiltration, number of epidermal layer and intercellular edema. Decrease in PMN was associated with low MPO activity and low oxidative stress in NDGA treated group. Application of TPA caused accumulation of inflammatory cells, which in turn led to excessive production of chemotactic cytokines. Cytokine IL-1, particularly IL-1*α* in mouse skin is involved in cell proliferation and hyperplasia in the tumor promotion stage [47]. Nakamura et al. [40] suggested that O_2_^−^ produced by the inflammatory cells play an important role in incessant and excessive production of chemotactic factors leading to chronic inflammation and hyperplasia.

This study of various markers of inflammation and oxidative stress of stage I tumor promotion supported by histological findings clearly indicates that NDGA has potential chemopreventive agent acting mainly by its anti-inflammatory and anti-oxidative properties. Although creosote bush lignan NDGA has a long history of safe use as an alternative medicine for topical uses in skin care system, its usage is limited by ingestion. In addition to NDGA, several other classes of compounds with therapeutic potential have been found in abundance in creosote bush. The result of the present study showed modulatory effects of one of its main active constituent NDGA against skin tumor promotion events substantiating the use of creosote bush in alternative therapies by the Native Americans and Mexicans.

Recently, several natural compounds having ability to intervene oxidative stress and inflammation have emerged as promising agents for cancer prevention [23–27]. For example, curcumin has a variety of positive pharmacological effects including cancer chemoprevention. Phase I human trials have also been performed using curcumin [48]. Salvioli et al. [48] proposed that besides cancer, curcumin is considered as a promising drug for the treatment of other diseases, most of which are related to aging process. Indeed curcumin has antioxidant and anti-inflammatory activity. Therefore, it could likely ameliorate other pathogenic conditions that share an inflammatory or oxidative pathway such as cardiovascular diseases, sporadic Alzheimer's disease (AD), sarcopenia, type II diabetes, arthrosis and arthritis. In view of promising results on curcumin, it may be appropriate to undertake clinical trials on NDGA, which has a similar mechanism of action. For this purpose, NDGA may be tested as a single candidate or in combination with other protective agents.

## 5. Conclusion

**Figure 6:**
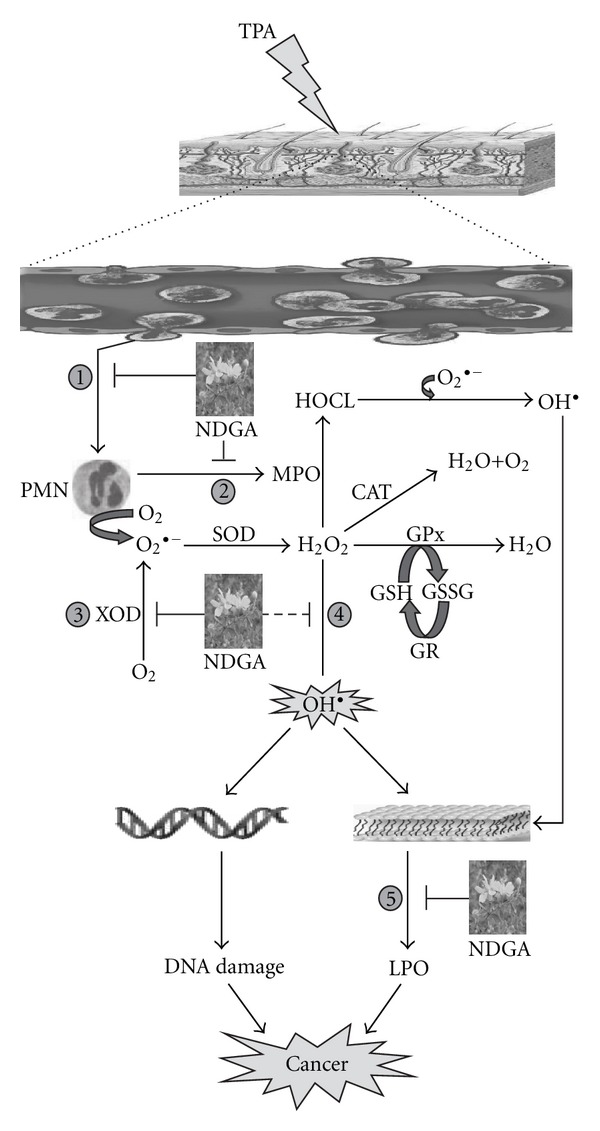
Possible pathways involving TPA induced generation of ROS and promising targets of NDGA. (1) NDGA prevents the TPA induced dermal infiltration and activation of PMNs. (2) NDGA downregulates MPO, one of the principal enzymes released upon PMN activation. MPO has been considered a key constituent of the PMNs cytotoxic armament by catalyzing the H_2_O_2_ dependent formation of HOCL, a potent oxidant. (3) It inhibits the activity of XO and thus prevents the formation of O_2_^•−^. This O_2_^•−^ is a substrate for the enzyme SOD, which catalyzes the formation of H_2_O_2_. H_2_O_2_ may be converted into highly reactive radical OH^•^. This OH^•^ may interact with lipid-rich plasma membrane and DNA molecule causes LPO and DNA damage that may result into tumor development. (4) NDGA may quench the hydroxyl radicals and thus prevents from deleterious effects. (5) NDGA also inhibits LPO. Confirmed (1–3 and 5) and unconfirmed intervention (4) are shown by unbroken and broken lines, respectively. TPA, 12-*O*-tetradecanoylphorbol-13-acetate; ROS, reactive oxygen species; NDGA, nordihydroguaiaretic acid; PMNs, polymorphonuclear leukocytes; MPO, myeloperoxidase; H_2_O_2_, hydrogen peroxide; HOCL, hypochlorous acid; XO, xanthine oxidase; SOD, superoxide dismutse; OH^•^, hydroxyl radical; LPO, lipid peroxidation.

A substantial database circumstantially implicates free radicals and ROS in tumor promotion [21]. Skin cells have developed a comprehensive set of antioxidant defense mechanism to prevent free radical formation and to limit their damaging effects [49]. Furthermore, inflammatory cells (neutrophils, macrophages, and eosinophils) are important endogenous sources of ROS [23]. In this study, we observed that double application of TPA is responsible for increase in oxidative stress and inflammation. As shown in Figure 6, application of TPA causes the infiltration of PMN at the site of exposure. TPA stimulates PMNs to undergo an oxidative burst that is characterized by rapid formation of O_2_^•−^ and H_2_O_2_. These infiltrated leukocytes have been shown to be major sources of reactive oxygen species generation. Also, PMNs activate oxidant-generating myeloperoxidase. MPO is the most abundant protein found in azeurophilic granule of PMN. Stimulation of PMN leads to degranulations, whereby the contents of the granules are released into the extracellular milieu causing cellular and tissue damage. Excessive production of reactive oxygen species due to massive activation of PMN resulting from TPA application can injure cellular bio-molecules such as nucleic acids, proteins, carbohydrates and lipids, which may leads to tumor development. As indicated in Figure 6 (target 1–5), NDGA afforded protection by intercepting at various stages of inflammation and oxidative stress, which makes it a potential candidate for skin cancer chemoprevention.

## Figures and Tables

**Table 1:** Anti-inflammatory effect of topically applied NDGA on TPA-induced morphological changes in skin as measured by number of epidermal layers, leukocyte infiltration and intercellular edema.

Group	Observation		
	No. of epidermal layers	Leukocyte infiltration	Intercellular edema
I (Ac/Ac)	2-3	−	−
II (Ac/TPA)	4–6	+++	+++
III [NDGA [15]/TPA]	3-4	+	+
IV [NDGA [25]/TPA]	3-4	+/−	−
V [NDGA [15]/Ac]	2-3	−	−
VI [NDGA [25]/Ac]	2-3	−	−

Leukocytes infiltration and intensity of edema are indicated by slight (+), moderate (++), severe (+++), equivocal (+/−) while (−) indicates absence of any significant change. Ac: acetone; NDGA: nordihydroguaiaretic acid; TPA: 12-*O*-tetradecanoylphorbol-13-acetate.
